# Noteworthy Literature of 2020: COVID Effects in Cardiac Surgery

**DOI:** 10.1177/10892532211012976

**Published:** 2021-05-14

**Authors:** Jessica Y. Rove, T. Brett Reece, Joseph C. Cleveland, Jay D. Pal

**Affiliations:** 1 University of Colorado, Aurora, CO, USA

**Keywords:** COVID-19, cardiac surgery, ECMO, aortic dissection, heart transplantation

## Abstract

COVID-19 has affected every aspect of life over the last year. This article reviews some of the effects that the pandemic had on cardiac surgery including volumes, ethical concerns with resource-intense procedures like dissection and transplant, and ECMO for COVID-19-derived refractory respiratory failure.

As physicians are well aware, the coronavirus disease-2019 (COVID-19) pandemic began affecting not only Americans with the infection but also patients with pathologies requiring cardiac procedures. Elective cardiac surgery was halted by both health department restrictions and the unknowns of the infectious potential of the virus in the first few months due to concerns of intensive care unit (ICU) bed and ventilator availability The health care system pivoted in unheard of directions to attempt to care for the initial surge of COVID-19-positive patients by halting all elective operations. In some hard-hit geographies, the elective surgical freeze extended into the fall and winter. As a result, cardiac surgical volumes throughout the country were decreased in 2020, complicated by concern that patients were deferring care due to fears of contracting the infection from emergency room or clinical visits. Despite these circumstances, heart transplantation continued to move forward with strict guidelines for infectious workups and processes to protect both the recipients and the caregivers. Emergency cardiac surgery continued for time-dependent pathologies including aortic dissections, endocarditis, and extracorporeal membrane oxygenation (ECMO) for cardiogenic shock. Many centers saw late presentations of myocardial infraction, heart failure, and even dissections, many to the degree that patients were no longer salvageable with operations that would have been straightforward during any other time. Even approaches to care provided was altered. Initial reports from Italy of ECMO for refractory oxygenation issues were on the verge of futile, but with experience, the role of ECMO evolved in many centers into lifesaving procedures for well-selected patients. This article will touch on each of these ideas in the review of noteworthy literature and events in 2020.

## Coronavirus Effect on Overall Cardiac Surgery Volume and Outcomes

The Society of Thoracic Surgeons Adult Cardiac Surgery Database provided the data for a landmark analysis exploring the effects of COVID-19 on cardiac surgery volumes and outcomes. Dr Thomas Nguyen and colleagues^1^ examined 2 large datasets asking the question of how COVID-19 affected cardiac surgery volumes and outcomes. The STS Adult Cardiac Surgery Database provided risk-adjusted data from January 1, 2018, to June 30, 2020. The Johns Hopkins COVID-19 Dashboard was similarly examined from February 1, 2020, to January 1, 2021. While the STS database yielded more than 700 000 cardiac surgery patients for analysis, the COVID-19 dashboard contained records from 20 million patients during this time.

The data yielded quite sobering findings. Overall, there was a 53% reduction in all cardiac surgery cases in the United States during this period. Furthermore, a 65% reduction in elective cases occurred. Even more striking was a regional variation in outcomes that followed. The mid-Atlantic region (New York, New Jersey, and Pennsylvania) assumed the greatest loss in case volume—a 71% decline. This region had historically performed better than expected with regard to outcomes. The region had enjoyed an observed to expected (O/E ratio) of 0.6 prior to COVID-19 for cardiac surgery mortality. The O/E ratio jumped by 167% for isolated CABG (coronary artery bypass graft) to 1.2 for patients operated on during the COVID-19 pandemic. The underlying explanation for this rapid increase in patient mortality is unable to be determined. Possible explanations include the following: patient-related factors, as many patients did not seek care for cardiovascular events and thus they presented with urgent/emergent situations; and potentially systems-based issues—ICU staff who were reassigned to care for COVID-19 patients, for example. The effects of cancellation or delay of elective cardiac surgery procedures during the COVID-19 pandemic will never be completely known, but these data offer insights into how the pandemic disrupted and directly contributed to excess patient mortality from cardiovascular disease during this period.

## Coronavirus Effects on Heart Transplant Volumes

Heart transplantation posed a unique programmatic challenge during the pandemic, in that some patients were often critically ill while awaiting a suitable donor heart. Others were stable outpatients, who were justifiably hesitant to being admitted to the hospital. Furthermore, with increasing numbers of patients being hospitalized with COVID-19 infections, a transplant patient would occupy an ICU bed for a prolonged period, which may be in short supply. In addition, despite best efforts to segregate patients and limit exposure, a COVID-19 infection in an immunosuppressed patient could be fatal. We will delve further into the logistic issues and the mechanism by which our institution addressed these challenges.

### Patient Factors

Patients who were listed at United Network for Organ Sharing (UNOS) Status 1 to 3 awaiting heart transplantation were considered emergent, and we proceeded with the logistic limitations as outlined below. UNOS Status 1 patients were most commonly being supported by ECMO, and therefore, transplant under these circumstances was justifiable. UNOS Status 2 patients were also supported by a nondischargeable mechanical circulatory support device, and had similar urgency as Status 1 patients. UNOS Status 3 encompasses patients with implantable left ventricular assist devices using 30 days of discretionary time, or patient supported with continuous inotropes. We elected not to use any discretionary time for a stable left ventricular assist device patient during this period. Patients who were deemed nondischargeable on inotropes remained hospitalized until a suitable donor organ was found. Patients awaiting transplant as UNOS Status 4 to 6 were outpatients, and we deferred these patients at the height of the pandemic.

### Logistical Factors

Given the need for social distancing to reduce potential exposure, our procurement team was reduced to 1 attending surgeon, 1 cardiothoracic surgical fellow, and 1 procurement coordinator. Medical students and other observing providers were not included on donor runs during this time period. In some circumstances, the donor hospital restricted outside personnel, in which case we utilized the donor heart if a local procurement surgeon was known to us, and available to perform the operation. In 3 instances, we were forced to decline the donor organ due to these factors.

**Figure 1.**
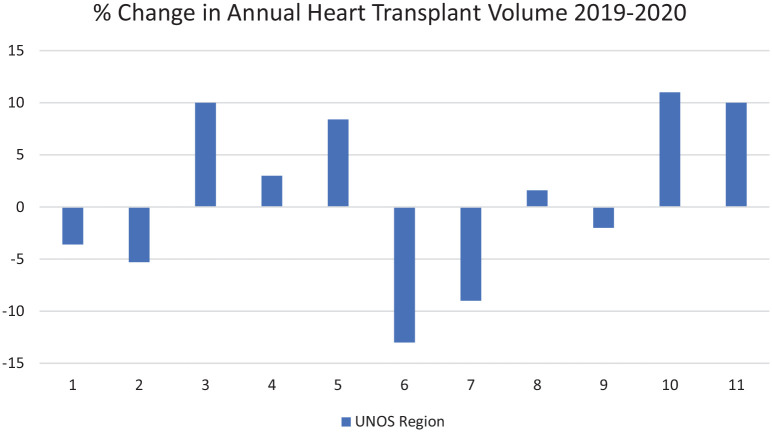
Effects of the pandemic on regional transplantation volumes.

During 2020, the national heart transplant volume increased by 3%, while our institutional volume decreased by 23%. Across the 11 UNOS regions, changes in annual volume ranged from −13% to 11% (Figure 1). While data do not exist to correlate these changes with state-wide restrictions, few centers were able to leverage the generalized reduction in hospital operations to increase transplant volumes. As the impact of the pandemic eased in Colorado in late 2020, elective operations were resumed, and we resumed accepting offers for all potential recipients.

## Ethics and Utilizations: Discussions Around Resource-Intense Procedures Like Dissection

The rapid rise of extended stay admissions, including ventilated needs, during the initial COVID-19 surge pressed the medical system on the potential for rationing care. As hospitals in New York dealt with conversion of rooms of all types into intensive care beds, the capability of the health system seemed to be on the verge of being stretched beyond its capability to offer care to all patients. As this limit approached, discussions regarding the viability of resource-intense operations like Type A dissections heated up. Many argued that indicators of complex aortic repairs and prolonged recovery would be prohibitive in the pandemic setting. This idea of rationing care would be unacceptable at any other time as operations are offered to all but the clearly futile patients.

Few can argue that mortality from emergent Type A repair is not significant. It is commonly reported between 10% and 30% following the operation. Hughes and colleagues^2^ attempted to address the idea of potential futility of operation in Type A dissection in a resource-restricted pandemic. They systematically reviewed literature regarding high-risk characteristics at presentation of Type A dissection that could lead to operational futility. While frailty and age are commonly discussed with regard to poor outcomes, the presentation of dissection does not frequently allow these assessments to be reliable or complete. As a result, the authors suggest that assessment of functional status should be discussed prior to proceeding with these repairs, but do not suggest that this alone should dictate rationing of care. Next, they pursued the impact of malperfusion syndrome on outcomes. Again, rather than declaring malperfusion to be an inoperable presentation, they suggest a paradigm for stable patients with malperfusion focusing on addressing ischemic beds first percutaneously with delayed formal dissection repair after malperfusion resolves. This would not apply to those with pericardial blood or other factors that may lead to early demise without formal repair. Expanding review of malperfusion presentations, the effects of cerebral malperfusion were explored. With limited data, they address only internal carotid occlusion and hemorrhagic strokes as relative contraindications to intervention in addition to dense stroke, which is commonly avoided in normal circumstances. Other situations addressed by the authors included novel anticoagulants, patient unwilling to take products, and complex presentations requiring transfer to aortic centers. Again the data remained limited such that no hard rationing advice could be given across the board short of sending these complicated situations to centers of excellence to vet the risk and benefits of surgery. They conclude that risk models do exist and that in the setting or limited resources, the discussion should be held with regard to futility of operation. Many of surgeons did see morbidly late presentation of dissections, but fortunately few centers had to deal with the ethical dilemmas of restricting care provided for these high-risk patients.

## ECMO Utilization for Coronavirus Patients With Refractory Respiratory Failure

ECMO shows promising survival benefit for select patients with COVID-19, but its impact on long-term recovery remains unknown. To determine the impact of ECMO on long-term outcomes of mechanically ventilated patients with COVID-19, we formed the Outcomes and Recovery After COVID-19 Leading to ECMO (ORACLE) Group, a collaborative between Johns Hopkins, University of Kentucky, Vanderbilt University Hospital, and University of Virginia, led by the University of Colorado.

The novel pathogen severe acute respiratory syndrome coronavirus 2 (SARS-CoV-2) triggering COVID-19 leads to invasive mechanical ventilation in an estimated 20% of hospitalized patients with an associated mortality as high as 80%.^1^ According to the international Extracorporeal Life Support Organization (ELSO), over 4700 patients with COVID-19 associated acute respiratory distress syndrome (ARDS) have been treated with ECMO.^2,3^ ELSO members report a 52% survival rate for patients with COVID-19 treated with ECMO, and factors affecting survival are being investigated at several centers.^3^

Survivors of critical illness are at high risk for long-term physical, psychological, and cognitive deficits. Postintensive care syndrome is a term used to describe the collective impairments in physical function, mental health, and cognition observed in ICU survivors. Follow-up of ARDS survivors including those who had influenza A subtype H1N1 or severe acute respiratory syndrome shows these deficits can persist for years and negatively impact meaningful recovery.^4-6^ In comparison to the extensive literature on post–intensive care syndrome after critical illness from ARDS, there are limited data on the specific long-term outcomes of ECMO survivors. Other than the 6-month follow-up in the United Kingdom–based multicenter CESAR trial, conclusions are largely limited to single-center investigations, small numbers of patients, and incomplete follow-up.^7,8^ ECMO survivors have been reported to experience decreased return to usual activity and worse chronic pain, in addition to depression, anxiety, and posttraumatic stress disorder (PTSD), which can persist up to 3 years after hospitalization.^7-9^ A single-center study from France reported 2-year follow-up demonstrating no difference in cognitive function, anxiety, depression, and PTSD between ARDS patients treated with ECMO compared with those who were not.^10^

ORACLE is a broadly multidisciplinary collaboration between 5 academic medical centers. Requirements for participating sites include (1) use of ELSO recommendations for ECMO support in patients with COVID-19 as a guideline for when and which patients to support with ECMO, (2) a specialized team for the management of ECMO patients, and (3) an established multidisciplinary post-ICU recovery clinic. ORACLE is evaluating survivors of COVID-19-related ARDS who required mechanical ventilation, including those who were supported with ECMO. Institutional review board waived the need for informed written consent (IRB#20-0731). Deidentified demographic, clinical, and laboratory data associated with the inpatient stay and ECMO course are being collected. All patients are referred for post-ICU recovery clinic follow-up during which period additional data collection includes objective quantitative measures of neuropsychiatric changes with the EQ-5D health questionnaire, anxiety, depression and PTSD screening, cognitive impairment with the Montreal Cognitive Assessment, and physical function with a 6-minute walk test, spirometry, and dyspnea score. These metrics are guided by the Core ICU Outcome Measurement Set for evaluating patients who recover from acute respiratory failure by an International Modified Delphi Consensus Study.^11,12,13^

Though discussion of our results is limited due to the peer-review process, preliminary data suggest that despite a more complex critical care course, patients who were treated with ECMO early in the COVID-19 pandemic did not have significantly worse long-term outcomes compared with survivors who were mechanically ventilated but did not receive ECMO. ORACLE is continuing to expand its partnerships with academic medical centers and is conducting analyses including determinants of positive long-term outcomes, ECMO survivorship, cost-effectiveness, and barriers to post-ICU recovery clinic follow-up.

## Conclusion

The last year has presented novel challenges on the health care system as a whole. The disruption of elective cases and the late presentations greatly altered the normality of cardiac surgery. Things like telehealth and remote care are most likely here to stay, but we hope to get back to our pre-COVID practices in the near future. The long-term effects of the pandemic remain to be seen on both our health care system and the patients we serve.
